# Influence of ancestry in mitochondrial processes in APOE4 carriers of Alzheimer's Disease

**DOI:** 10.1002/alz70855_104961

**Published:** 2025-12-24

**Authors:** Katrina Celis, Maria Muniz, Farid Rajabli, Patrice G Whitehead, Karen Nuytemans, Liyong Wang, Daniel A. Dorfsman, Eileen H Bigio, Marsel Mesulam, David A. A. Bennett, Theresa Schuck, Sandra Weintraub, Changiz Geula, Camille Alba, Clifton L. Dalgard, David A. Davis, William K. Scott, Jonathan L Haines, Derek M. Dykxhoorn, Margaret Pericak‐Vance, Anthony J Griswold, Juan I Young, Jeffery M Vance

**Affiliations:** ^1^ John P. Hussman Institute for Human Genomics, University of Miami Miller School of Medicine, Miami, FL, USA; ^2^ John P. Hussman Institute for Human Genomics, Miller School of Medicine, University of Miami, Miami, FL, USA; ^3^ Dr. John T. Macdonald Foundation Department of Human Genetics, University of Miami Miller School of Medicine, Miami, FL, USA; ^4^ University of Miami Miller School of Medicine, Miami, FL, USA; ^5^ Northwestern University, Chicago, IL, USA; ^6^ Mesulam Center for Cognitive Neurology & Alzheimer's Disease, Chicago, IL, USA; ^7^ Rush Alzheimer's Disease Center, Chicago, IL, USA; ^8^ Department of Pathology and Laboratory Medicine, Institute on Aging and Center for Neurodegenerative Disease Research, The Perelman School of Medicine at the University of Pennsylvania, Philadelphia, PA, USA; ^9^ Northwestern ADC Neuropathology Core, Northwestern University Feinberg School of Medicine, Chicago, IL, USA; ^10^ Henry M. Jackson Foundation for the Advancement of Military Medicine, Bethesda, MD 20817, Bethesda, MD, USA; ^11^ The American Genome Center, Center for Military Precision Health, Uniformed Services University of the Health Sciences, Bethesda, MD, USA; ^12^ Brain Endowment Bank, Department of Neurology, University of Miami Miller School of Medicine, Miami, FL, USA; ^13^ John P. Hussman Institute for Human Genomics, University of Miami, Miami, FL, USA; ^14^ Department of Population and Quantitative Health Sciences, Case Western Reserve University School of Medicine, Cleveland, OH, USA; ^15^ John P. Hussman Institute for Human Genomics, Miller School of Medicine, Miami, FL, USA

## Abstract

**Background:**

*APOE*e4 is the strongest genetic factor for Alzheimer disease (AD). The risk associated with the *APOEe4* allele differs across ancestries with African ancestry (AF) showing lower AD risk compared to European ancestry (EU). Multiple cell types and biological processes may be contributing to the differences in *APOE*e4 risk seen between AF and EU individuals. To determine the role of mitochondria in the ancestry‐biased different risk, we sought to investigate if mitochondrial processes (MtFx) associated with AD were influenced by ancestry in AD *APOEe4* carriers.

**Method:**

Single nuclei RNA sequencing (snRNA‐seq) was performed on frontal cortex from 17 homozygous AD *APOEe4* carriers with either EU Local Ancestry (EU‐LA; *N* = 10) or AF Local Ancestry (AF‐LA; *N* = 7). Data were analyzed by comparing gene expression differences in AF vs EU samples. MitoXplorer was used to identify differential mitochondrial processes, while REACTOME facilitated the building of regulatory networks.

**Result:**

Cells highly expressing *APOE* like astrocytes, microglia, and vascular leptomeningeal cells (VLMCs) show an increased expression of both nuclear and mitochondrial encoded mitochondrial (MT) genes in EU‐LA vs AF‐LA samples. Specifically, we observed an increase in expression of pro‐apoptotic, oxidative phosphorylation, and mitophagy MT‐genes in EU samples. We identified the transcriptional regulator *PPARG* as a likely mediator of *APOEe4* effects in these cells.

**Conclusion:**

Our ancestry‐specific analysis of snRNA‐seq in AD provides a unique overview into the underlying changes in mitochondrial processes in *APOEe4* AD carriers. This study suggests that ancestry differences in mitochondrial processes might underly the AD risk difference observed for EU and AF populations. Whether these transcriptional differences in mitochondrial gene expression are associated with the higher levels in APOE expression seen in EU individuals is still unknown but subject of future research.

